# Surgery and Surgery Approach Affect Survival of Patients With Stage I-IIA Small-Cell Lung Cancer: A Study Based SEER Database by Propensity Score Matching Analysis

**DOI:** 10.3389/fsurg.2022.735102

**Published:** 2022-02-11

**Authors:** Xiaolu Chen, Jia-Li Zhu, Huaying Wang, Wanjun Yu, Tao Xu

**Affiliations:** ^1^Department of Respiratory and Critical Care, The Affiliated People's Hospital of Ningbo University, Ningbo, China; ^2^School of Medicine, Tongji University, Shanghai, China

**Keywords:** small cell lung cancer, I-IIA, surgery, SEER database, propensity score matching

## Abstract

**Purpose:**

The purpose of this study was to observe the significance of surgery and its approach in stage I-IIA (according to 8th American Joint Committee on Cancer Staging Manual) small-cell lung cancer (SCLC) using the Surveillance, Epidemiology, and End Results (SEER) database.

**Patients and Methods:**

A total of 1,421 patients from ages 31 to 93 years who were diagnosed with stage I-IIA SCLC in the SEER database from 2010 to 2015 were analyzed. The 1:1 propensity score matching analysis was used to minimize the effect of selection bias, and 355 pairs of patients' data was performed subsequent statistical analysis. K–M analysis and a Cox proportional hazards model were used to observe the role of surgery and other clinical features in the patients' prognoses on cancer-specific survival (CSS).

**Results:**

Overall, within the whole cohort, the 3- and 5-year CSS rates were 41.0 and 34.0%, respectively. In a Cox regression that adjusted for other clinical features, patients were more likely to benefit from the surgery [hazard ratio (HR) 0.292, 95% confidence interval (CI) 0.237–0.361, *P* < 0.001]. Unadjusted 5-year cancer-specific survival among those with surgery was 55.0%, compared with 23.0% among those without surgery. In the propensity scored-matched dataset, however, 5-year CSS among those with surgery was 54.0%, compared with 17.0% among those without surgery (HR 0.380, 95%CI 0.315–0.457, *P* < 0.001). In patients who received surgery, cases with lobectomy had a better 5-year CSS than those without lobectomy (65.0 vs. 39.0%). The lobectomy might be a protective factor for patients who underwent resection in CSS (HR 0.433, 95%CI 0.310–0.604, *P* < 0.001).

**Conclusions:**

We suggested that the surgery and lobectomy were the independent prognostic as well as the protective factors in stage I-IIA SCLC patients. We recommended that patients with no surgical contraindications receive surgery, preferably, lobectomy.

## Introduction

Lung cancer is mainly divided into non-small cell lung cancer (NSCLC) and small cell lung cancer (SCLC), which still ranks second in the global cancer spectrum morbidity, and first in mortality. In the United States alone in 2021, however, it is estimated that the number of new cases will reach 235,760, and the death toll will reach 131,800 (1). About 13% of patients with lung cancer fall into the small cell lung cancer category. Because of high cellular proliferation and early metastatic spread, the 5-year overall survival rate for SCLC was about 6% which was still low (2). The advanced SCLC patients were mainly treated with chemotherapy and radiotherapy (3); however, for early SCLC, studies had shown that patients can benefit from surgery (4–7).

Some studies demonstrated that 5-year survival rates were approximately 40 to 60% for patients who underwent surgery in the early-stage SCLC, such as stage I, (8, 9). According to the 7th American Joint Committee on Cancer (AJCC) Staging Manual (10), the American Society of Clinical Oncology (ASCO) guidelines (11), and the National Comprehensive Cancer Network (NCCN) guidelines (3) stage I-IIA SCLC patients with received surgery with adjuvant chemotherapy. The 8th edition of the AJCC Staging Manual was enacted on January 1, 2017. There were some differences between the 7th and 8th editions (12) as per Jiro Okami's study (13) which suggested that the 5-year overall survival (OS) with stage IA in the 8th edition was better than that in 7th edition; however, the 5-year OS with IB and IIA in 8th was worse than that in 7th edition. For the early-stage SCLC in the 8th edition, the significance of surgery and surgical methods for survival remains to be explored.

We extracted data from the database of the Surveillance, Epidemiology, and End Results (SEER), including stage T1-4N0M0 SCLC patients who confirmed diagnosis during 2010–2015, according to 7th AJCC. We got the data of a total of 1,991 patients. We transformed their combined stage to the 8th edition. Eventually, 1,421 patients with stage IA-IIA were included in this study. We have used the early-stage SCLC patients recorded in the database of SEER, a population-based cohort of 1,421 patients, to observe the significance and the approach of surgery.

## Methods

### Patients

This study includes all the patients who were diagnosed with a histologically confirmed SCLC from January 2010 to December 2015. Histology and site of disease were coded in SEER according to the International Classification of Diseases (ICD) for Oncology, Edition 3 (ICD-O-3). Patients who met the following criteria were enrolled in the study: (1) patients with SCLC (ICD-0-3 histology code 8041/3, 8043/3, 8044/3, 8045/3, and 8073/3); (2) pathologically confirmed patients in stage IA-IIA according to 8th edition of AJCC Staging Manual (we translated the 7th edition of the staging into the 8th edition based on the patients' medical record); (3) patients with tumor in the main bronchi and lung (ICD-0-3 site code c34.0-c34.9) were collected for this study. Patients with the following standards were excluded. (1) those in stage IIB-IIIA and (2) who only received pathological anatomy. All patient records were anonymized before analysis. The information we collect in the SEER database includes patients' ID, age at diagnosis, gender, laterality, approach of surgery, chemotherapy situation, radiation situation, race, tumor differentiation, tumor location, pTNM stage, tumor extension, survival months, survival status, cancer causes of disease (COD), and the situation of pleura invasion and lymph nodes. Eventually, the study collected the information on 1,421 patients.

### Treatment

According to the record of SEER database, the main approaches of treatment include chemotherapy, radiation (radioactive implants, radioisotopes, beam radiation and combination of beam with implants or isotopes), and surgery. There were many kinds surgery recorded, such as lobectomy, sublobectomy (wedge and segmental resection), and interventional therapy (tumor destruction: excision, laser ablation, and cautery).

### Follow-Up

The collected patients had a clear survival time and survival status. We regarded cancer-specific survival (CSS) as our observation endpoint. The CSS was from the date of diagnosis to the time of death caused by SCLC. Follow-up time ranged from 0.0 to 83.0 months, with an average of 21.7 ± 0.52 months.

### Statistical Analysis

Statistical analysis was performed using SPSS Statistics 25.0 software (IBM SPSS, Inc., Chicago, IL, USA), R version 3.5.2 and Graph pad Prism 8. Hazard risk (HR) with 95% confidence intervals (95% CIs) were calculated by multivariate regression analysis. Unadjusted associations between clinical features and outcomes were displayed using Kaplan–Meier curves and compared using the log–rank test. A multivariable proportional hazards regression model was used to determine the association between surgery and other clinical features. A nomogram was shows the results of regression analysis. A *P* value < 0.05 was considered to be statistically significant. Statistical tests were based on a two-sided significance level. Similarly, the Kaplan–Meier analysis and the log–rank tests were used to compare survival curves between groups. To minimize the effect of other clinical factors, we used TNM stage, tumor location, laterality, age, gender, race, whether to receive radiation or/and chemotherapy, tumor differentiation, and first malignant as matching variables. To maximize execution performance and randomize case order when drawing matches, we used the propensity score matching analysis with a match tolerance of 0.02 by the SPSS 25.0 for clarity. After matching, the balance between the groups was checked via the χ^2^ test. Cases were censored at death or the end of follow-up. The selection of CSS as a primary clinical end point was considered to be most clinically relevant.

## Results

### Characteristics of Patients

The clinical characteristics of patients in the study cohort are listed in Table 1. Among the 1,421 patients, 654 (46.0%) were me, 767 (64.0%) were women, 1,237 (87.1%) belonged to the white race, 129 (9.1%) belonged to the black race, and 55 (3.9%) belonged to other races. The patients' age ranged from 31 to 93 years (median, 71.0 years). In the whole cohort, the 3- and 5-year CSS rates were 41.0 and 34.0%, respectively, and the median and mean times from diagnosis to the last censoring date were 19.0 and 24.2 months, respectively. Follow-up time ranged from 0.0 to 83.0 months, with an average of 21.7 ± 0.52 months.

**Table 1:** The characteristics and the results of K–M analysis in limited SCLC patients.

		**CSS (months)**		
**Variables**	**No. of**	**Median**	**95% CI**	***P*-Value**
	**patients (%)**			
**Sex**				0.053
Male	654 (46.0%)	28.0	23.0–33.1	
Female	767 (54.0%)	32.0	26.3–37.7	
**Age at diagnosis (years)**				**<0.001**
≤ 65	411 (28.9%)	55.0	41.4–68.6	
>65	1010 (71.1%)	26.0	23.3–28.7	
**Race**				**0.001**
White	1237 (87.1%)	31.0	26.4–35.6	
Black	129 (9.1%)	27.0	13.0–41.0	
Other	55 (3.9%)	18.0	13.3–22.7	
**Surgery**				**<0.001**
No	972 (68.4%)	22.0	19.7–24.3	
Yes	449 (31.6%)	NA	NA	
**Radiation**				**0.003**
No	697 (49.0%)	24.0	19.6–28.4	
Yes	724 (51.0%)	33.0	28.2–37.8	
**Chemotherapy**				**<0.001**
No	488 (34.3%)	22.0	18.3–25.7	
Yes	933 (65.7%)	35.0	29.6–40.4	
**Tumor differentiation**				**<0.001**
Grade I	11 (0.8%)	62.0	0.0–135.5	
Grade II	22 (1.5%)	23.0	0.0–48.6	
Grade III	283 (19.9%)	58.0	NA	
Grade IV	292 (20.5%)	38.0	29.8–46.2	
Unknown	813 (57.2%)	24.0	21.1–26.9	
**Laterality**				**<0.001**
Right	823 (57.9%)	33.0	27.1–38.8	
Left	596 (41.9%)	28.0	23.6–32.4	
Unknown	2 (0.1%)	0.0	NA	
**TNM stage**				**<0.001**
IA	951 (66.9%)	35.0	28.4–41.6	
IB	285 (20.1%)	23.0	17.3–28.7	
IIA	185 (13.0%)	18.0	12.0–24.0	
**Tumor Location**				**0.006**
Upper lobe	833 (58.6%)	30.0	24.3–35.7	
Middle lobe	86 (6.1%)	56.0	29.7–82.4	
Lower lobe	410 (28.9%)	30.0	23.9–36.1	
Main bronchi	48 (3.4)	18.0	14.5–21.5	
Overlapping lesion	7 (0.5%)	43.0	NA	
Other	37 (2.6%)	21.0	9.8–32.2	

*The meaning of the bold values was significant difference statistically*.

Within the cohort, the number of patients who received surgery was 449 (31.6%); the remaining patients were 972 (68.4%) (without surgery) (Table 1). The main (pathological tumor node metastasis) pTNM stage was IA (*N* = 951, 66.9%) and IB (*N* = 285, 20.1%). In this cohort, the tumor was located in the upper lobe in some patients (*N* = 833, 58.6%) and in the lower lobe in others (*N* = 410, 28.9%). Among the degrees of tumor differentiation, there were 11 (0.8%) well-differentiated, 22 (1.5%) moderately differentiated, 283 (19.9%) poorly differentiated, 292 (20.5%) undifferentiated, and a part of tumor differentiation (*N* = 813, 57.2%) was unknown.

### The Results of K–M Analysis in All Variables

In this study, patients were divided into groups according to their classification, respectively (Table 1). We found the survival curve had a better stratification effect in the groups of age at diagnosis, race, tumor differentiation, laterality, tumor location, pTNM stage, and the situation of surgery, chemotherapy, and radiation. The *p* values were all < 0.05.

### The χ^2^ Test of Propensity Scored-Matched Dataset

We used the propensity score matching analysis to minimize the effect of the TNM stage to receive radiation or/and chemotherapy and age at diagnosis (≤65 or >65) on CSS. In the propensity scored-matched dataset, there were 355 pairs of patients and we found that the *p* value of χ^2^ test after matching was different from primary dataset (Table 2). We suggested that the results of matching minimized the effects of other factors.

**Table 2:** The clinicopathological characteristics of patients before matching and propensity scored-matched patients.

	**Before matching**			**After matching**		
**Variables**	**Surgery-no**	**Surgery-yes**	***P* value**	**Surgery-no**	**Surgery-yes**	***P*-Value**
	**(*N* = 970)**	**(*N* = 451)**		**(*N* = 355)**	**(*N* = 355)**	
**Sex**			0.788			0.880
Male	445 (68.0%)	209 (32.0%)		155 (49.7%)	157 (50.3%)	
Female	527 (68.7%)	240 (31.3%)		200 (50.3%)	198 (49.7%)	
**Age (years)**			**<0.001**			0.870
≤ 65	239 (58.2%)	172 (41.9%)		107 (49.5%)	109 (50.5%)	
>65	732 (72.5%)	278 (27.5%)		248 (50.2%)	246 (49.8%)	
**Radiation**			**<0.001**			0.806
No	353 (50.6%)	344 (49.4%)		247 (49.7%)	250 (50.3%)	
Yes	619 (85.5%)	105 (14.5%)		108 (50.7%)	105 (49.3%)	
**Chemotherapy**			0.058			1.00
No	318 (65.2%)	170 (34.8%)		145 (50.0%)	145 (50.0%)	
Yes	654 (70.1%)	279 (29.9%)		210 (50.0%)	210 (50.0%)	
**Laterality**			0.100			0.314
Right	546 (66.3%)	277 (33.7%)		185 (51.7%)	173 (48.3%)	
Left	424 (71.1%)	172 (28.9%)		112 (47.5%)	124 (52.5%)	
Other	2 (100%)	0 (0.0%)		NA	NA	
**TNM stage**			**<0.001**			0.603
IA	579 (60.9%)	372 (39.1%)		281 (50.2%)	279 (49.8%)	
IB	232 (81.4%)	53 (18.6%)		56 (51.9%)	52 (48.1%)	
IIA	161 (87.0%)	24 (13.0%)		18 (42.9%)	24 (57.1%)	

*The meaning of the bold values was significant difference statistically*.

### Univariate and Multivariate Analyses

Before matching, univariate and multivariate analyses were performed to identify correlations between clinical characteristics and CSS. As shown in Table 3, univariate analyses identified the following clinical characteristics as significant CSS prognostic factors in patients with SCLC: age at diagnosis, other race, the grade III of tumor differentiation, the unknown grade of tumor differentiation, tumor location with main bronchi, pTNM stage, whether to receive surgery, chemotherapy, or radiation. Further multivariate analysis based on those characteristics confirmed that only age at diagnosis, other race, whether to receive surgery, pTNM stage, the grade III of differentiation, whether to receive chemotherapy, and whether to receive radiation were independent prognostic factors (Table 3). Our study revealed that these factors are significantly associated with prognosis in stage I-IIA SCLC patients.

**Table 3:** Univariate and multivariate Cox regression analysis for cancer-specific survival in patients with stage I-IIA SCLC cancer (Cox regression's method is Forward: LR).

	**Univariate analysis**			**Multivariate analysis**		
	**HR**	**95% CI**	***P*-Value**	**HR**	**95% CI**	***P*-Value**
**Before matching**						
**Sex**						
Male/Female	0.864	0.746–1.004	0.056			
**Age at diagnosis (years)**						
≤ 65/>65	1.559	1.312–1.852	**<0.001**	1.373	1.151–1.637	**<0.001**
**Race**						
White	0.821	0.663–1.017	0.071			
Black	0.980	0.752–1.277	0.879			
Other	1.818	1.310–2.522	**<0.001**	1.880	1.350–2.618	**<0.001**
**Surgery**						
No vs. yes	0.380	0.315–0.457	**<0.001**	0.292	0.237–0.361	**<0.001**
**Radiation**						
No vs. yes	0.800	0.690–0.929	**0.003**	0.478	0.402–0.567	**<0.001**
**Chemotherapy**						
No vs. yes	0.701	0.600–0.818	**<0.001**	0.794	0.672–0.938	**0.007**
**Tumor differentiation**						
Grade I	0.410	0.132–1.276	0.124			
Grade II	1.145	0.661–1.982	0.629			
Grade III	0.626	0.510–0.786	**<0.001**	0.806	0.654–0.994	**0.044**
Grade IV	0.864	0.716–1.043	0.129			
Unknown	1.510	1.294–1.761	**<0.001**	NA	NA	0.807
**Laterality**						
Right	0.890	0.766–1.034	0.128			
Left	1.124	0.967–1.306	0.128			
Unknown	NA	NA	NA			
**TNM stage**						
IA vs. IB vs. IIA	1.298	1.177–1.431	**<0.001**	1.217	1.098–1.348	**<0.001**
**Tumor Location**						
Upper lobe	0.967	0.832–1.124	0.663			
Middle lobe	0.726	0.514–1.025	0.069			
Lower lobe	0.993	0.843–1.171	0.938			
Main bronchi	1.713	1.207–2.431	**0.003**	NA	NA	0.173
Overlapping lesion	0.540	0.135–2.163	0.384			
Other	1.445	0.954–2.190	0.083			
**After matching**						
**Sex**						
Male/Female	0.758	0.610–0.940	**0.012**	0.721	0.580–0.897	**0.003**
**Age at diagnosis (years)**						
≤ 65/>65	1.506	1.180–1.923	**0.001**	1.364	1.063–1.751	**0.015**
**Race**						
White	0.754	0.547–1.039	0.084			
Black	1.038	0.685–1.573	0.859			
Other	1.896	1.192–3.015	**0.007**	2.013	1.256–3.226	**0.004**
**Surgery**						
No vs. yes	0.322	0.256–0.404	**<0.001**	0.287	0.228–0.363	**<0.001**
**Radiation**						
No vs. yes	0.591	0.462–0.757	**<0.001**	0.580	0.440–0.764	**<0.001**
**Chemotherapy**						
No vs. yes	0.667	0.537–0.829	**<0.001**	0.745	0.588–0.944	**0.015**
**Tumor differentiation**						
Grade I	0.552	0.177–1.721	0.306			
Grade II	0.995	0.493–2.006	0.988			
Grade III	0.609	0.460–0.806	**0.001**			
Grade IV	0.895	0.694–1.155	0.394			
Unknown	1.575	1.268–1.956	**<0.001**			
**Laterality**						
Right	0.946	0.760–1.177	0.618			
Left	1.057	0.849–1.316	0.618			
Unknown	NA	NA	NA			
**TNM stage**						
IA vs. IB vs. IIA	1.011	0.836–1.221	0.913			
**Tumor Location**						
Upper lobe	1.111	0.891–1.385	0.251			
Middle lobe	0.439	0.247–0.781	**0.005**	0.538	0.302–0.959	**0.035**
Lower lobe	0.959	0.754–1.221	0.734			
Main bronchi	1.597	0.935–2.729	0.087			
Overlapping lesion	0.437	0.061–3.111	0.408			
Other	1.773	0.972–3.235	0.062			

*The meaning of the bold values was significant difference statistically*.

After matching, we found that age at diagnosis, other race, sex, the grade III of tumor differentiation, the unknown grade of tumor differentiation, tumor location with middle lobe, and whether to receive surgery, chemotherapy, or radiation were associated with the CSS in patients. In the multivariate analysis, sex (HR 0.721, 95%CI 0.580–0.897, *P* = 0.003), age at diagnosis (HR 1.364, 95%CI 1.063–1.751, *P* < 0.001), other race (HR 2.013, 95%CI 1.256–3.226, *P* = 0.004), whether to receive surgery (HR 0.287, 95%CI 0.228–0.363, *P* < 0.001), whether to receive chemotherapy (HR 0.745, 95%CI 0.588–0.944, *P* = 0.015), whether to receive radiation (HR 0.580, 95%CI 0.440–0.764, *P* < 0.001) and middle lobe (HR 0.538, 95%CI 0.302–0.959, *P* = 0.035) were confirmed as independent prognostic factors (Table 3).

**Figure 1:**
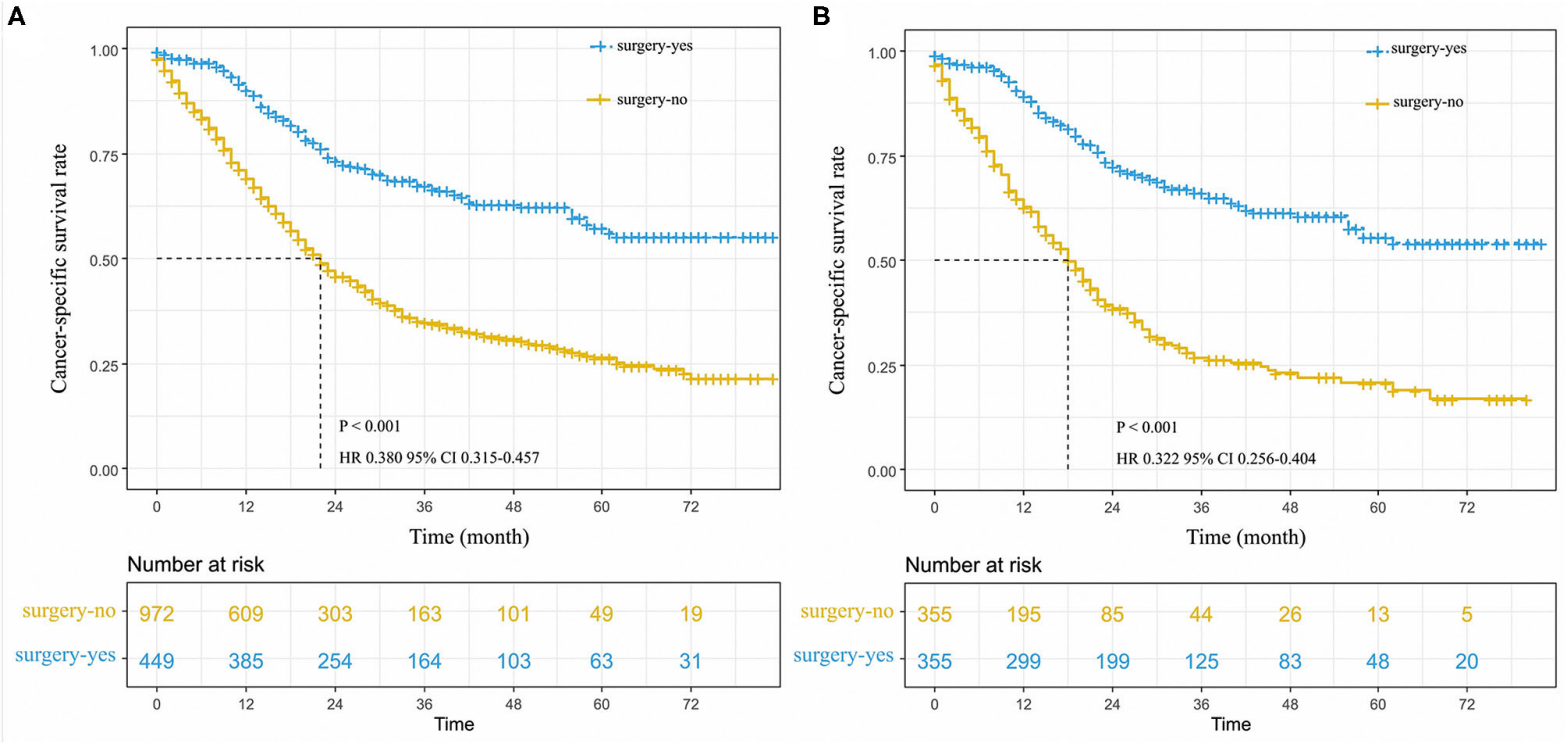
Cancer-specific survival curve for stage I-IIA small cell lung cancer patients with according to the treatment approach in the unmatched cohort **(A)**, and matched patients **(B)**.

**Figure 2:**
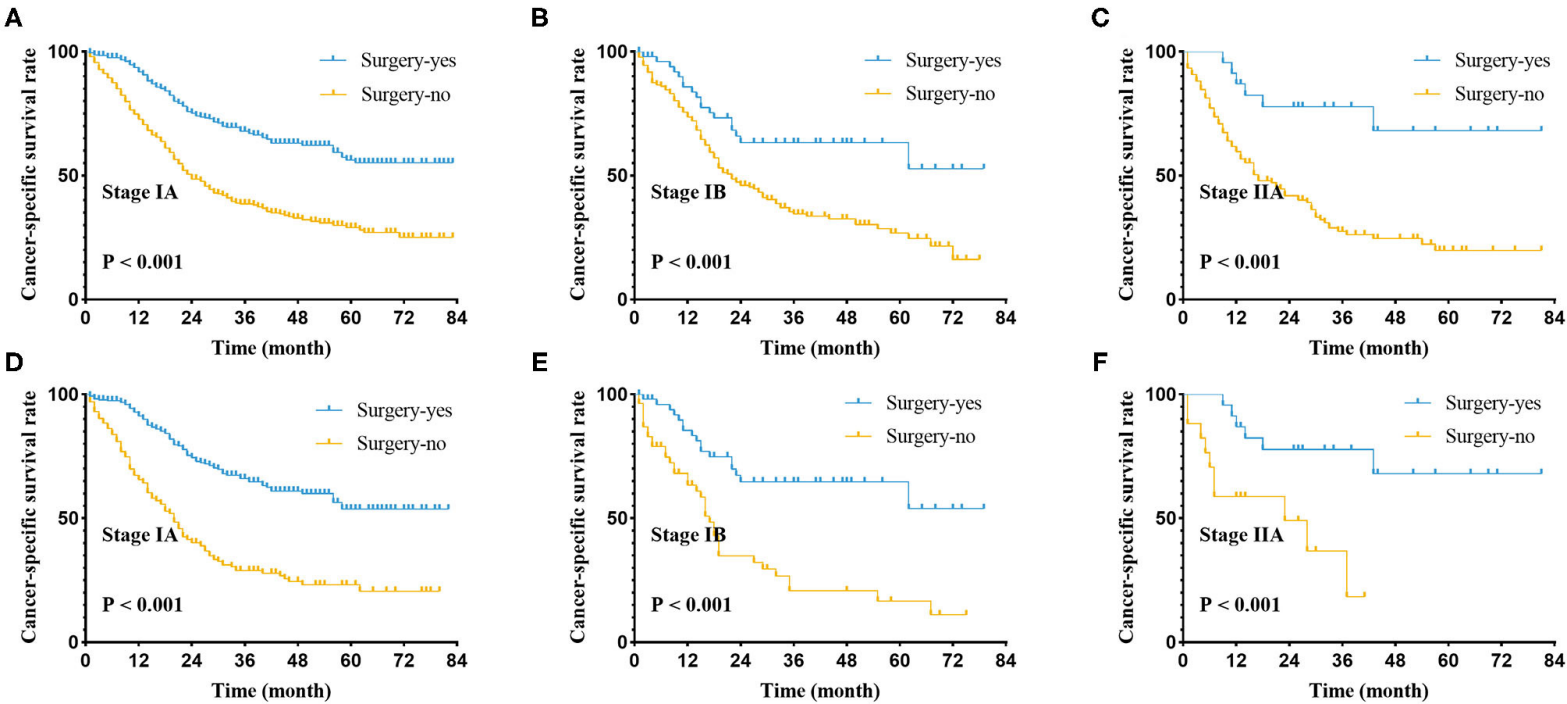
Cancer-specific survival curve for stage I-IIA small cell lung cancer patients with according to the treatment approach in the unmatched cohort **(A–C)** and matched patients **(D–F)** of pathological stage.

**Figure 3:**
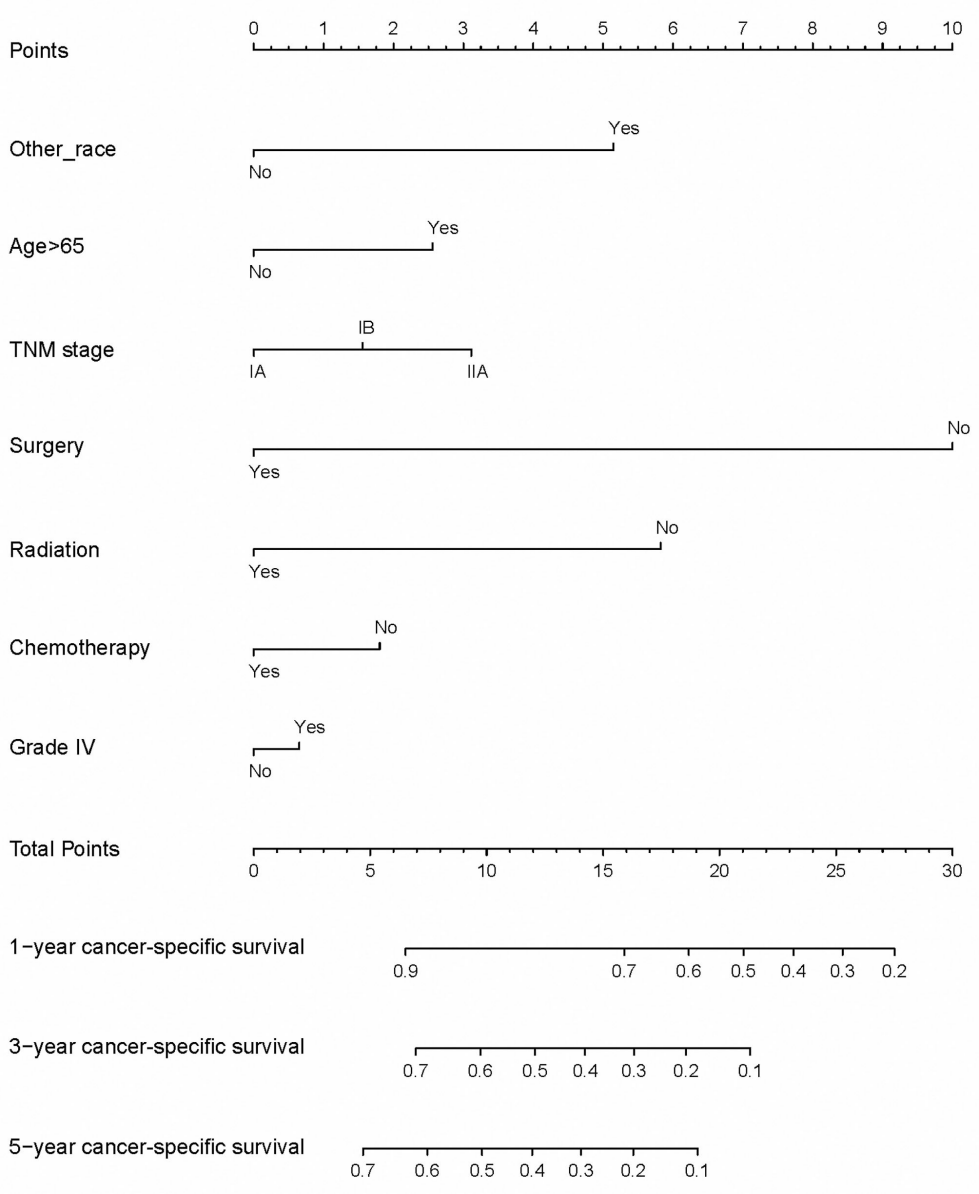
A nomogram is to show the results of multivariate regression.

We suggested that surgery was an independent prognostic factor in SCLC, which acted as a protective factor, which might indicate a better survival rate (Figure 1). We conducted a classification analysis in stage IA-IIA and found surgery could improve the survival rates of SCLC patients in every stage (Figure 2, all *P* < 0.001). Besides, a nomogram was used to show the results of multivariate regression before matching (Figure 3). The surgery played the most important role in affecting prognosis for those patients.

### The Significance of Surgery Approach in SCLC Patients Receiving Resection

**Figure 4:**
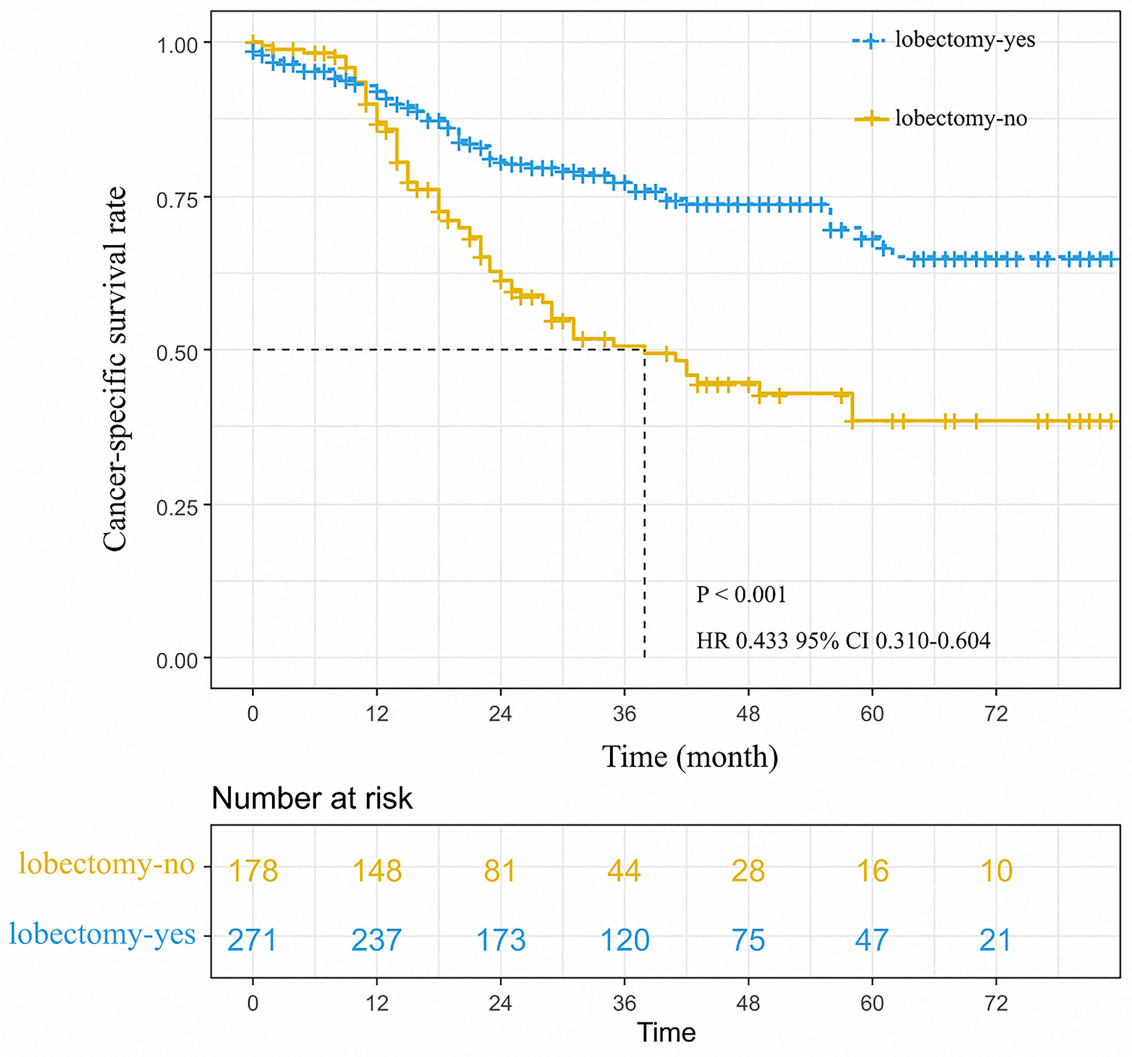
Cancer-specific survival curve for small cell lung cancer patients according to the surgical approach.

We screened patients of stage I-IIA SCLC who underwent surgery from the recruits. We found that 449 patients had received surgery and analyzed their data. There were 271 (60.1%) and 172 (38.1%) patients received lobectomy and sublobectomy, respectively (Table 4). The age at diagnosis and lobectomy were considered to be independent prognostic factors in SCLC patients undergoing surgery by univariate and multivariate analysis (Table 5). The lobectomy of surgery approach was a protective factor and an indicator of better survival (Figure 4). We also compared all approaches of surgery with significance of survival in early-stage SCLC patients, and the results suggested that lobectomy was better than other approaches (Supplementary Figure 1).

**Table 4:** The characteristics in the SCLC patients with stage I-IIA who received surgery (*N* = 449).

**Variables**	**No. of patients (%)**	**Variables**	**No. of patients (%)**
**Age at diagnosis (years)**		**Sex**	
≤ 65	172 (38.3%)	Male	209 (46.5%)
>65	277 (61.7%)	Female	240 (53.5%)
**Race**		**Chemotherapy**	
White	412 (91.8%)	No	170 (37.9%)
Black	24 (5.3%)	Yes	279 (62.1%)
Other	13 (2.9%)	**Laterality**	
**Surgery Approach**		Right	277 (61.7%)
Lobectomy	271 (60.4%)	Left	172 (38.3%)
Sub lobectomy	172 (38.3%)	**Radiation**	
Pneumonectomy	4 (0.9%)	No	344 (76.6%)
Other surgery	2 (0.4%)	Yes	105 (23.4%)
**Tumor differentiation**		**Tumor Location**	
Grade I	8 (1.8%)	Upper lobe	282 (62.8%)
Grade II	12 (2.7%)	Middle lobe	32 (7.1%)
Grade III	147 (32.7%)	Lower lobe	125 (27.8%)
Grade IV	147 (32.7%)	Main bronchi	3 (0.7%)
Unknown	135 (30.1%)	Overlapping lesion	3 (0.7%)
**TNM stage**		Other	4 (0.9%)
IA	372 (82.9%)		
IB	53 (11.8%)		
IIA	24 (5.3%)		

**Table 5:** Univariate and multivariate Cox regression analysis for cancer-specific survival in patients with stage I-IIA SCLC who received surgery (Cox regression's method is Forward: LR).

	**Univariate analysis**			**Multivariate analysis**		
	**HR**	**95% CI**	***P*-Value**	**HR**	**95% CI**	***P*-Value**
**Sex**						
Male/Female	0.699	0.502–0.973	**0.034**	NA	NA	0.065
**Age at diagnosis (years)**						
≤ 65/>65	1.587	1.113–2.262	**0.011**	1.597	1.120–2.276	**0.010**
**Race**						
White	1.003	0.542–1.857	0.992			
Black	0.758	0.335–1.717	0.507			
Other	1.572	0.643–3.839	0.321			
**Surgery Approach**						
Lobectomy	0.433	0.310–0.604	**<0.001**	0.462	0.330–0.645	**<0.001**
Sub lobectomy	2.200	1.578–3.068	**<0.001**	NA	NA	0.383
Pneumonectomy	1.115	0.275–4.497	0.882			
Other surgery	NA	NA	NA			
**Radiation**						
No vs. yes	0.799	0.534–1.196	0.275			
**Chemotherapy**						
No vs. yes	0.683	0.489–0.955	**0.026**	NA	NA	0.621
**Tumor differentiation**						
Grade I	0.627	0.155–2.532	0.512			
Grade II	1.342	0.549–3.276	0.519			
Grade III	0.848	0.593–1.214	0.368			
Grade IV	1.434	1.023–2.010	**0.036**	NA	NA	0.327
Unknown	0.806	0.552–1.176	0.263			
**Laterality**						
Right	0.884	0.631–1.240	0.476			
Left	1.131	0.807–1.585	0.476			
Unknown	NA	NA	NA			
**TNM stage**						
IA vs. IB vs. IIA	0.986	0.723–1.346	0.931			
**Tumor Location**						
Upper lobe	1.411	0.992–2.006	0.055			
Middle lobe	0.302	0.112–0.815	**0.018**	0.369	0.136–1.004	0.051
Lower lobe	0.819	0.561–1.196	0.301			
Main bronchi	2.165	0.535–8.751	0.279			
Overlapping lesion	1.065	0.149–7.624	0.950			
Other	2.362	0.752–7.420	0.141			

*The meaning of the bold values was significant difference statistically*.

### Cancer-Specific Survival

On the whole, unadjusted 3- and 5-year cancer-specific survival among those who received surgery was 63.0 and 55.0%, respectively, compared with 31.0 and 23.0% among those without surgery (HR 0.380, 95%CI 0.315–0.457, *P* < 0.001; Figure 1A). The median survival time of patients without surgery was 22 months; however, there was no median survival time in patients who received surgery. In a Cox regression that adjusted for age, other race, pTNM stage, the grade III of differentiation, whether to receive chemotherapy or radiation, tumor location of main bronchi, and unknown differentiation (Table 3), patients were more likely to benefit from surgery compared with those without surgery (Figure 1A).

In the propensity scored-matched dataset, we found that 3- and 5-year CSS had an evident difference between two groups of patients who received surgery and without surgery, respectively. The 3- and 5-year CSS among those with surgery were 61.0 and 54.0%, compared with 24.0 and 17.0% among those without surgery (*P* < 0.001, Figure 1B). The median survival time of patients without surgery was 18 months, however, there was no median survival time in patients that received surgery. After minimizing the effect of other clinical factors, the results of 1:1 match analysis revealed that patients with surgery had an increasing advantage of survival than those without surgery in the early-stage SCLC (HR 0.292, 95%CI 0.237–0.361, *P* < 0.00, Table 3; Figure 1B).

In the cohort which SCLC patients received surgery, we found that unadjusted 3- and 5-year cancer-specific survival among those that received lobectomy was 74.0 and 65.0%, respectively, compared with 45.0 and 39.0% among those without lobectomy (HR 0.433, 95%CI 0.310–0.604, *P* < 0.001; Figure 4). The median survival time of patients without lobectomy was 38.0 months; however, there was no median survival time in patients who received lobectomy. In a Cox multivariate regression, patients that received lobectomy had a better survival HR 0.462, 95%CI 0.330–0.645, *P* < 0.001, Table 5).

## Discussion

The occurrence and development of SCLC is complex, and the decision about treatment still requires multidisciplinary participation. For advanced SCLC, patients were mainly treated with chemotherapy and radiotherapy (3, 11, 14, 15); however, for early SCLC, studies had shown that patients can benefit from surgery (4, 16–21). Because SCLC is prone to recurrence and metastasis, systemic therapy remains dominant throughout the treatment of SCLC. Some research studies have suggested that patients with stage I-IIA SCLC could receive surgery. As the launch of 8th edition of AJCC, the role of surgery in limited SCLC needs to be reassessed. A part of patients with stage IB and IIA in the 7th edition of AJCC drifted to stage IIA and IIB of the 8th edition, respectively. In patients undergoing surgery, the choice of surgical approach is also worthy of attention by clinicians.

This study aimed to provide useful information to support clinicians' decisions. During this research, the patient's clinical information was analyzed, including the indicators shown in Table 1. We found that age at diagnosis, other race, whether to receive surgery, pTNM stage, the grade III of differentiation, whether to receive chemotherapy, and whether to receive radiation were independent prognostic factors. We used propensity score matching to minimize the effect of other variables on CSS (22). After balancing these factors, we suggested that the influence of surgery on survival in patients with stage I-IIA was more precise. Through this and other studies, we compared the survival advantage in different surgical approaches (Supplementary Figure 1), and suggested that the surgery and lobectomy were the independent prognostic and protective factors in limited SCLC. If there were no surgical contraindications, we recommended that patients with limited SCLC should receive lobectomy. We also have found the importance of early screening in small cell lung cancer. For patients of SCLC with stage T1-2N0M0, accurate judgment of the disease before surgery makes it possible to undergo surgery as soon as possible to improve the prognosis.

This study has some limitations. First, the retrospective study used the SEER database wherein the distribution of ethnic groups is not balanced. Second, it is recommended that data from other regions be included in this study to make the results more generalized. Third, in limited SCLC, this study had not analyzed the role of surgery in multidisciplinary treatment (chemotherapy, radiation and so on), and the number of patients who received pneumonectomy or tumor destruction was too small. Fourth, only patients with stage I-IIA SCLC were enrolled. We suggest that further prospective research is necessary to confirm our findings.

## Data Availability Statement

The raw data supporting the conclusions of this article will be made available by the authors, without undue reservation.

## Ethics Statement

The studies involving human participants were reviewed and approved by Ethics Committee of Ningbo Yinzhou People's Hospital. The Ethics Committee waived the requirement of written informed consent for participation.

## Author Contributions

WY conceptualized the study. XC, HW, and TX curated the data. XC, J-LZ, and HW did the formal analysis. HW and TX designed the methodology and validated the study. XC and WY were involved in project administration and supervision. XC and HW wrote the original draft. XC, HW, TX, and WY were involved in writing, reviewing, and editing. XC and J-LZ revised the manuscript. All authors have read and agreed to the published version of the manuscript.

## Funding

This work was supported by 2017 Zhejiang Provincial TCM Prevention and Treatment Plan for Major Diseases (Item No. 2018ZY010).

## Conflict of Interest

The authors declare that the research was conducted in the absence of any commercial or financial relationships that could be construed as a potential conflict of interest.

## Publisher's Note

All claims expressed in this article are solely those of the authors and do not necessarily represent those of their affiliated organizations, or those of the publisher, the editors and the reviewers. Any product that may be evaluated in this article, or claim that may be made by its manufacturer, is not guaranteed or endorsed by the publisher.
