# Supplementary figures and images for: Surgery and Surgery Approach Affect Survival of Patients With Stage I-IIA Small-Cell Lung Cancer: A Study Based SEER Database by Propensity Score Matching Analysis
Source: Front Surg. 2022 Feb 11;9:735102. doi: 10.3389/fsurg.2022.735102 (PMC8878678; doi:10.3389/fsurg.2022.735102)

Supplementary Fig1 The survival curve of different surgical methods in limited SCLC.

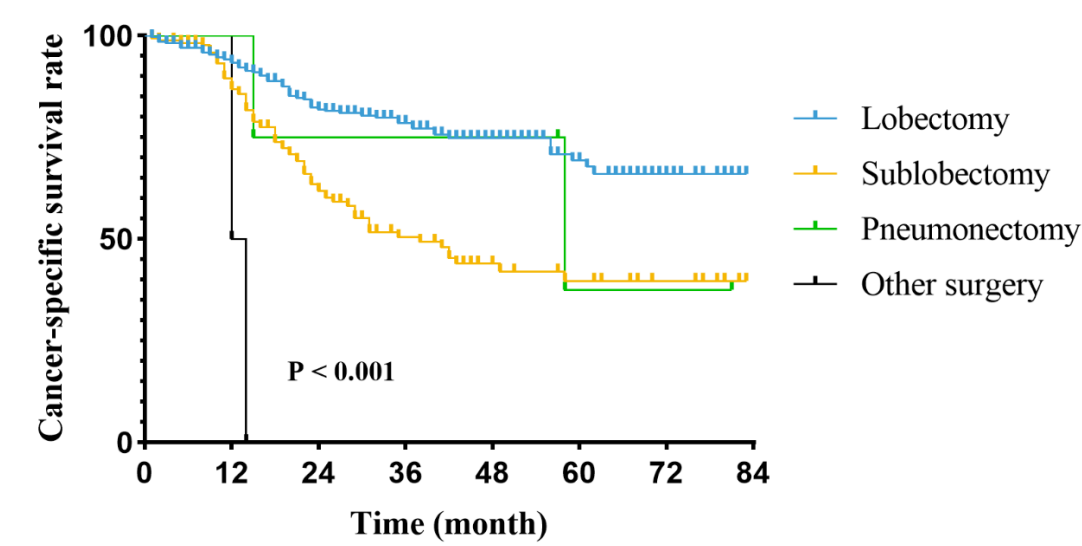

Supplement: Supplementary file 1 [file Image_1.pdf]
